# Tunable up-conversion single-photon detector at telecom wavelengths

**DOI:** 10.1515/nanoph-2022-0528

**Published:** 2022-11-07

**Authors:** Jin-Woo Chae, Jin-Hun Kim, Youn-Chang Jeong, Yoon-Ho Kim

**Affiliations:** Department of Physics, Pohang University of Science and Technology (POSTECH), Pohang 37673, South Korea; Department of Physics, Pohang University of Science and Technology (POSTECH), Pohang 37673, South Korea

**Keywords:** frequency up-conversion, quantum communication, single-photon detector, sum-frequency generation, telecom C band

## Abstract

Up-conversion single-photon detectors (UCSPD) are based on sum-frequency generation of the telecom band single-photons to near-infrared wavelengths at which efficient and low-noise silicon single-photon detectors are available. Moreover, because of high dynamic range of silicon single-photon detectors, UCSPD is suitable for high-speed quantum communication. UCSPDs reported to date, however, have a very narrow fixed window of detectable wavelengths, severely limiting their applications in wavelength-multiplexed quantum networks. In this work, we report a tunable UCSPD module that covers the complete telecom C band, making it suitable for quantum communication networks based on sharing wavelength-multiplexed entangled photons.

## 1 Introduction

Single-photons and entangled-photons are often exploited as long-distance information carriers in quantum communication, encoding qubits or qudits in a quantum superposition of various photonic degrees of freedom, e.g., polarization, frequency, time-bin, and orbital angular momentum [1], [2], [3], [4], [5]. For long-distance applications, similarly to classical optical communication, photons in the telecom C-band wavelengths are used to exploit the low attenuation (∼0.2 dB/km at 1550 nm) of the deployed optical fiber network.

Unlike classical fiber optic communication, where erbium fiber amplifiers are readily available to amplify the attenuated optical pulses, amplifiers cannot be used in quantum communication as arbitrary quantum information cannot be copied due to the no-cloning theorem. Thus, in long-distance fiber-optic quantum communication, robust and high signal-to-noise ratio single-photon detection at the telecom band is critical. However, this is a rather challenging technical problem compared to detecting near-infrared single photons at which reliable and low-noise silicon single-photon detectors (SPDs) are readily available. Typically, single-photon detection at the telecom band has been done with InGaAs SPDs. The small footprint and the ease of operation of InGaAs SPDs are attractive in quantum communication experiments, but these detectors suffer from a high dark count rate, necessitating a long dead time, thus reducing the maximum count rate to about 100 kHz (ensuring the detection linearity requires to further cap the count rate at a few tens of kHz) [6]. The dark count rate of semiconductor single-photon detectors can be reduced by operating them at cryogenic temperatures [7], [8], [9], [10], [11], but lowering the operating temperature comes with increased afterpulsing effects, limiting the lowest-possible operating temperatures (typical silicon SPDs operate at −20 °C, and typical InGaAs SPDs operate at −80 °C) [12, 13]. Recently, superconducting nanowire SPDs have entered the market for telecom band single photon detection [14], [15], [16], but the detection system is not rack-mountable and requires constant cryogenic cooling, and most importantly very costly, although they offer superior performance.

An alternative approach is up-conversion single-photon detector (UCSPD). The principle of UCSPD is to convert telecom photons into near-infrared photons through a sum-frequency generation (SFG) process [17] and measure them via a typical Si-based single-photon counting module (SPCM) operating at visible wavelength. The UCSPD detects the telecom photons with a high quantum efficiency (∼70% at 700 nm) and a low dark count rate (∼100 Hz) with a short dead times (∼20 ns) [6], which are the superiorities of Si single-photon avalanche photodiodes compared to InGaAs single-photon avalanche photodiodes. In the past two decades, UCSPDs have been actively researched in various directionality, such as wavelength tunability [18], high detection efficiency [19], high coupling efficiency [20], ultra-low noise [21], and integrated module [22]. However, to maximize UCSPDs’ performance, telecom photons’ wavelengths measured with UCSPDs are fixed, and even the tunable UCSPD has a narrow detectable bandwidth of 5 nm.

In this paper, we present a tunable UCSPD that covers the complete telecom C band. Our UCSPD makes use of the type-0 SFG process to convert the incoming telecom C-band single photons into the wavelength of 836 nm for detection with a silicon SPCM. The detection bandwidth of our UCSPC is determined to be 80 GHz, which is compatible with the dense wavelength division multiplexing (DWDM) components. Note that the broad detectable range can be achieved by using a tunable optical parametric oscillator (OPO) pump laser combined with the phase-matching condition of the SFG process.

## 2 Quantum frequency converter based on sum-frequency generation

Our tunable UCSPD consists of the OPO for pump laser generation and an SFG unit based on waveguide periodically poled lithium niobite (PPLN). This section will describe the quantum frequency converter (QFC) module’s structure and operation principle. Then, we present QFC performance measured with a tunable continuous-wave (CW) laser.

**Figure 1:**
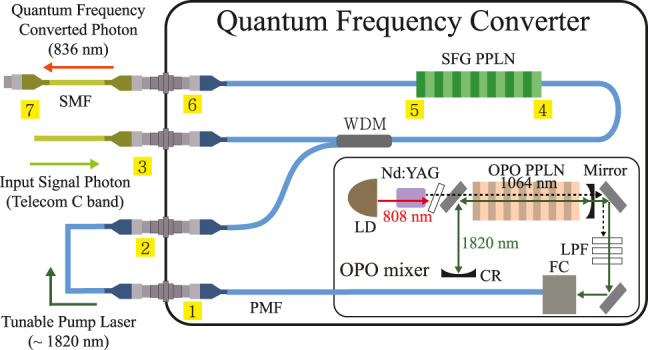
The structure of the quantum frequency converter (QFC) based on the sum-frequency generation (SFG) process. The laser diode of the optical parametric oscillator (OPO) mixer generates 808 nm light (red), converting it into 1064 nm (dashed) at the Nd:YAG crystal. This light produces both the light near 1820 nm (green) and 2560 nm by the OPO processes. The 1064 nm light is filtered through long-pass filters, and the 2520 nm light is intrinsically filtered from the 1550 nm fiber. The pump light generated near 1820 nm is combined with the telecom photons through the wavelength-division multiplexing (WDM) and transmitted to the periodically poled lithium niobite (PPLN) waveguide for generating the 836 nm converted photons by an SFG process. The numbers in the yellow square boxes are the path information for Table 1. LD: laser diode, SFG PPLN: SFG PPLN (waveguide), OPO PPLN: OPO PPLN (bulk), CR: cavity reflector, SMF: single-mode fiber, PMF: polarization-maintaining fiber.

Figure 1 shows the structure of the QFC where the photon conversion process takes place in our UCSPD. Our QFC consists of two main parts; the OPO mixer for the tunable wavelength pump laser and the SFG PPLN (poling period: 24.8 μm, length 49.5 mm) for the SFG process. First, the OPO mixer can generate the pump light from 1775 to 1840 nm by adjusting the temperature of the OPO PPLN (poling period: 31.97 μm, crystal length: 50 mm). This tunable pump wavelength enables all C-band photons to be detected. Second, the SFG PPLN always converts the telecom photons into 836 nm with various correlated pump wavelengths by the type-0 SFG process. Table 1 represents the transmission measurement results of all components.

**Table 1:** Transmissions of the quantum frequency converter components.The connection information is shown in Figure 1.

1820 nm		Transmission
Connection	Description	
1 to 2	FC/APC connection	0.945
2 to 4	WDM + FC/APC connection	0.67
4 to 5	Waveguide coupling + propagation	0.496
**1550 nm**		**Transmission**
**Connection**	**Description**	
3 to 4	WDM + FC/APC connection	0.89
4 to 5	Waveguide coupling + propagation	0.436
**836 nm**		**Transmission**
**Connection**	**Description**	
5 to 6	Waveguide surface coupling	0.773
6 to 7	FC/APC connection	0.87

Next, we report the key performance of the QFC. Since our UCSPD was designed to detect all C-band photons, the optimal PPLNs’ temperatures are investigated to satisfy the phase-matching condition of the SFG process. Here, the optimal temperatures are selectively examined for detecting nine DWDM-compatible wavelengths, which are properly defined by the center wavelengths of DWDM international telecommunication union (ITU) channels (35, 37, 39, 41, 43, 45, 47, 49, and 51) to be used for the final single photon experiment.

**Figure 2:**
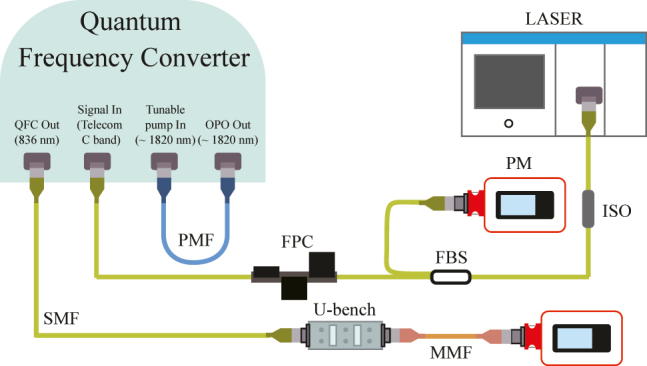
Schematic diagram of the optical setup for QFC performance test at the high input power regime. FPC: fiber polarization controller, PM: power meter, ISO: isolator, FBS: fiber beam splitter, U-bench: U-bench with band-pass filters, SMF: single-mode fiber, PMF: polarization-maintaining fiber, MMF: multi-mode fiber.

Figure 2 shows the experimental setup to measure the key performance of the QFC using the tunable CW laser (NetTest, Osics ECL 1560). To find the optimized PPLNs’ temperature sets for maximizing the SFG conversion efficiency, after setting one telecom wavelength among the nine wavelengths, we varied the two PPLNs’ temperatures and defined the optimized temperature sets. We identified the nine temperature sets of the PPLNs for the nine telecom wavelengths and tested the performance under those conditions. However, during the frequency up-conversion processes, the high-intensity pump laser naturally generates Raman and parametric noises. Therefore, we precisely designed the SFG processes to convert all telecom photons into 836 nm photons and filtered out them using the customized band-pass filters with a bandwidth of 5 nm centered at 836 nm. These two band-pass filters for noise reduction at around 836 nm were installed on the U-bench, which is placed after the QFC. Through this setup, we obtained a detectable range and a bandwidth by varying the telecom wavelength using the tunable laser after setting the optimized nine temperature sets of the PPLNs.

**Figure 3:**
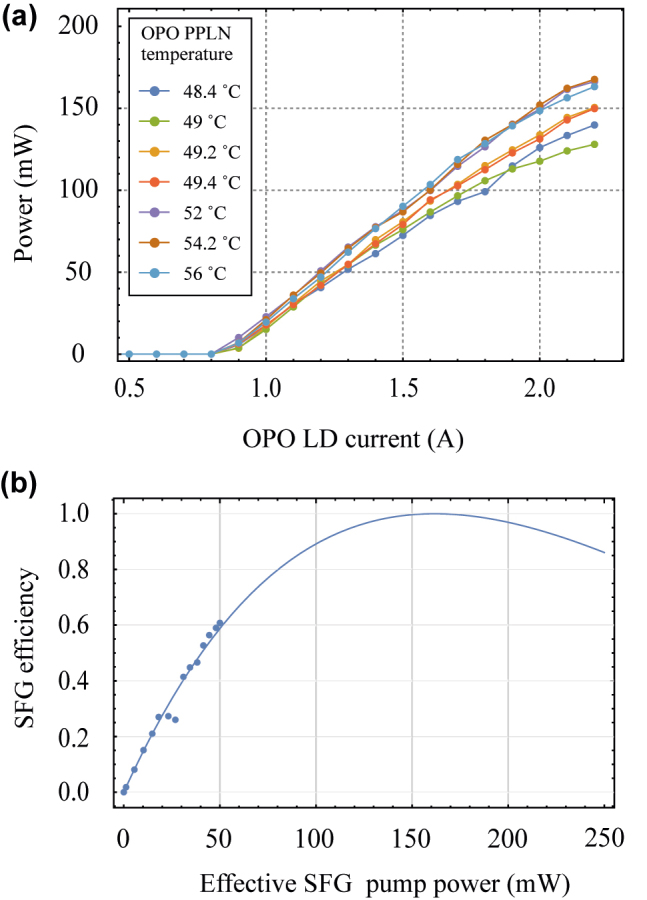
Quantum frequency converter performance test results using the tunable laser. (a) Pump power measured by port 2 in Figure 1 according to laser diode (LD) current at the seven OPO PPLN temperatures. (b) SFG conversion efficiency as a function of the effective SFG pump power (effective SFG pump power = pump power × coupling efficiency from Figure 1, 2 to 5).

Figure 3 presents the key performance of our UCSPD. Figure 3(a) shows the OPO pump power measured at port 2 in Figure 1 while raising the OPO laser diode current according to the seven optimized temperatures of the OPO PPLN, including two overlapped temperatures for the nine temperature sets. The power of the OPO pump measured by port 2 in Figure 1 linearly increases as the OPO laser diode current increases from the threshold current of 0.8 A to the maximum current of 2.2 A. To increase an SFG conversion efficiency, a sufficient pump power is necessary. The sufficient pump has been analyzed by [23]

$$\eta_{\text{SFG}}=\frac{N_{\text{c}}\left(L\right)}{N_{\text{s}}\left(0\right)}={\text{sin}}^{2}\left(\alpha L\sqrt{P_{\text{pump}}}\right),$$

using *P*_pump_ to represent the pump power before it enters the waveguide, and considering the power coefficient of *α*^2^, the power inside the waveguide is *P*_*p*_ = *α*^2^*P*_pump_, where *L* is the waveguide length and *N*_c_(*L*) is the converted signal photon number at the end of the waveguide, and *N*_s_(0) is the input signal photon number at the entrance of the waveguide. However, since we experimented at the classical level, the photon number should be converted to power. When we use $\text{N}=\frac{\text{P}}{\hbar \omega }$ , we can express the SFG conversion efficiency by

(1) $$\eta_{\text{SFG}}=\frac{P_{\text{c}}}{P_{\text{s}}}\frac{\omega_{\text{s}}}{\omega_{\text{c}}}={\text{sin}}^{2}\left(\alpha L\sqrt{P_{\text{pump}}}\right),$$

where *P*_c_ is the converted signal power at the end of the waveguide where *L* is the waveguide length, *P*_s_ is the input signal power at the entrance of the waveguide, and *ω*_s_ and *ω*_c_ represents the angular frequency of the input signal and the converted signal. When the OPO laser diode current is set to the maximum current of 2.2 A, the OPO pump power is 128–167 mW depending on the OPO PPLN’s temperatures. Figure 3(b) shows the SFG conversion efficiency measurement result to detect 1546.12 nm with the OPO PPLN’s temperature of 49.4 °C and the SFG conversion efficiency theoretical curve in Eq. (1). The SFG conversion efficiency is the value where the SFG conversion efficiency becomes maximized for sufficient pump power. From the results, we confirmed that our OPO pump power is insufficient to satisfy the maximum SFG conversion efficiency because of the structural restrictions of our OPO cavity.

**Figure 4:**
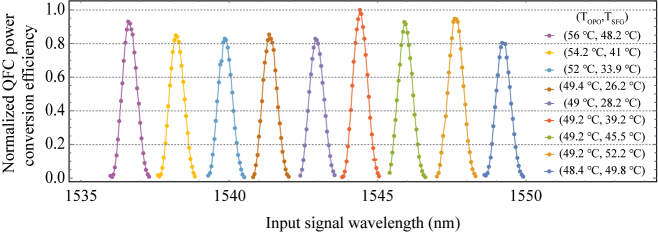
Normalized QFC power conversion efficiency (QFC power conversion efficiency = output converted light power at 836 nm/input telecom C-band light power) measurement results according to input signal wavelength with the nine optimized PPLNs’ temperature sets (*T*_OPO(SFG)_ represents the OPO(SFG) PPLN temperature); all QFC power conversion efficiencies are normalized to the maximum QFC power conversion efficiency when measuring 1544.4 nm.

Figure 4 shows the normalized QFC power conversion efficiencies and the detectable bandwidths according to the various input signal wavelengths. As the quasi-phase matching condition is changed according to each signal wavelength, the optimized PPLNs’ temperature setting should be done priorly at the UCSPD to detect the signal wavelength. Therefore, we set the nine temperature sets of the PPLNs optimized at the nine center wavelengths and measured the conversion efficiencies while varying the signal wavelength near each center wavelength. According to the results, we can confirm that the conversion efficiencies are nearly kept constant over the 14 nm region, which means our UCSPD will have the detectable range of at least 14 nm with 80 GHz detectable bandwidth.

## 3 Performance of the tunable up-conversion single-photon detector

As the performance tests of the QFC have been confirmed only at the classical level, it is necessary to confirm the characteristics of the UCSPD, which appears only at the single-photon level. In this section, we will show two experiments using an attenuated laser and a heralded single-photon source at the single-photon level and discuss their results.

### 3.1 Detection of the attenuated telecom C-band laser at the single-photon level

The difficulty of single-photon experiments is that noises not measured at the classical level have a dominant effect at the single-photon level. Similarly, reducing the dark count rate caused by unexpected noises is crucial for developing an SPD. The main source of the dark counts generated in UCSPDs is the Raman noise caused by the pump laser, as the UCSPDs measure single-photon signals using the SFG processes with the high-intensity pump laser. When approximately 10^7^ photons are incident on the medium, about one Raman-noise photon is generated [24]. These small noises are not measurable at the classical level but have a dominant effect at the single-photon level. Therefore, we characterized the Raman noise in our system using an attenuated laser to measure the dark counts generated by the Raman noise under various conditions. Furthermore, it can be confirmed whether the detection efficiency estimated by measuring the performance of the QFC in the previous section is the same even in the single-photon level experiment.

**Figure 5:**
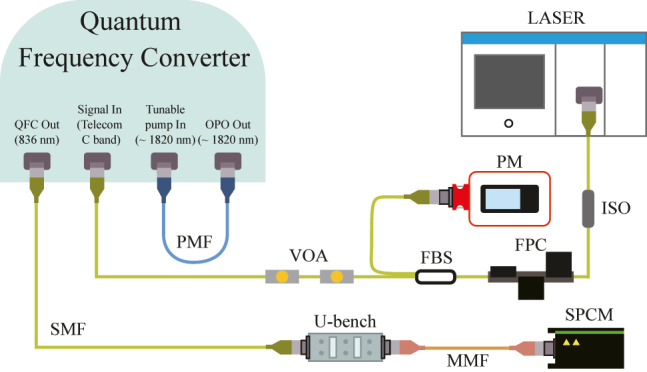
The schematic diagram of the optical setup for up-conversion single-photon detector (UCSPD) performance test using an attenuated laser. VOA: variable optical attenuator, SPCM: Si single-photon counting module.

Figure 5 shows the single-photon level experiment scheme using the attenuated laser. We set the signal input counts to 100 kHz using two 55 dB variable optical attenuators with the tunable CW laser. Also, the power meter was replaced with the Si SPCM (Excelitas, SPCM-800-13-FC) to measure the single photons. The Si SPCM has a quantum efficiency of 60% at 836 nm and a dark count rate of ∼180 Hz. First, in order to check how much Raman noise is generated in which condition, we measured the Raman noise for six temperature settings of the OPO PPLN while increasing the SFG PPLN temperature from 30 to 60 °C. As the Raman noise generates from the high-intensity pump laser, we only connected the OPO pump laser operated at the maximum OPO laser diode current (2.2 A) and measured the Raman noise. In our UCSPD, the prominent Raman noise is generated while the second-harmonic generation light of the OPO pump laser passes through the PPLNs. It cannot be filtered out by the 836 nm band-pass filters. According to previous research on PPLN [19], a Raman shift probability of PPLN exponentially decreases after 880 cm^−1^ (in our UCSPD system, when OPO PPLN temperature is set to 43 °C, the 880 cm^−1^ Raman shift of the second-harmonic generation light is identical with 836 nm). Therefore, to avoid the Raman noise, we needed to check how far away from this point the Raman noise can be suppressed sufficiently.

**Figure 6:**
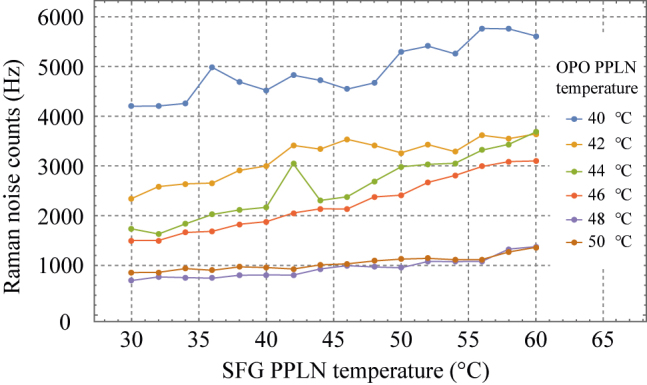
The Raman noises measurement results according to the SFG PPLN temperature with the six OPO PPLN’s temperature settings.

Figure 6 shows the measurement results of the Raman noises. The Raman noise counts increase while increasing the temperature of the SFG PPLN; however, the order of the Raman noise is entirely dependent on the temperature of the OPO PPLN. The Raman noise can be suppressed to about 1 kHz when the OPO PPLN’s temperature is set above 47 °C.

**Figure 7:**
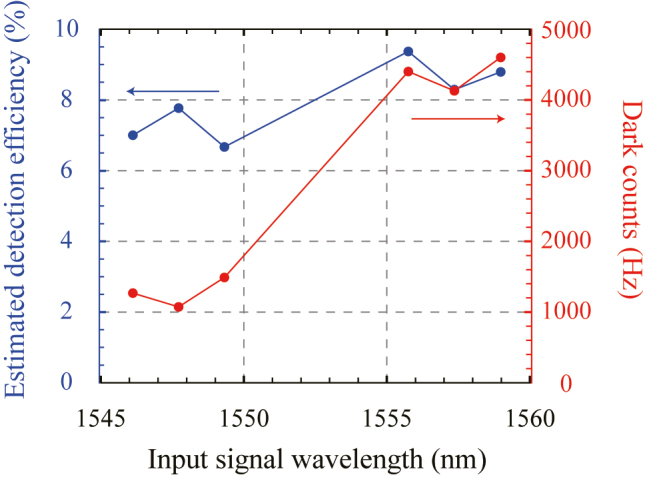
Detection efficiency and dark counts measurement results with the six wavelengths of the telecom photon.

Next, to confirm where the Raman noise order drops sharply, we experimented with the six optimized temperature sets of the PPLNs for the 6-wavelength telecom photons between 1545 nm and 1560 nm and measured their Raman noises. The used wavelengths are the center wavelengths of DWDM ITU channels 23, 25, 27, 35, 37, and 39. To estimate detection efficiency, we set the power level of the tunable CW laser to 100 kHz single photon level and experimented. In addition, to measure the dark count, as in the previous Raman experiment, only the OPO pump laser operating at 2.2 A was input to measure the dark count. Figure 7 is the result of measuring the detection efficiencies and the dark count rate of the UCSPD. When checking the dark count rate measurement results, it can be seen that the Raman noises of about 1 kHz are generated when measuring the signals shorter than 1550 nm. Therefore, to guarantee low dark counts of about 1 kHz, our UCSPD can be used to measure single photons below 1550 nm. The measurement results show the detection efficiencies from 6.7 to 9.4% because the required conditions, such as pump power and SFG conversion efficiency, varied according to the signal wavelength.

These values can also be estimated based on the QFC performance identified in the previous section. The estimated channel efficiencies of all processes of the UCSPD are shown in Table 2. When we calculate a total detection efficiency with all channel efficiencies, we can define the estimated total detection efficiency of 8.14%. Although we had obtained the experimental detection efficiencies of 6.7–9.4% depending on the SFG’s various phase matching conditions, we concluded that these results are consistent with the estimated detection efficiency of 8.14%.

**Table 2:** Channel efficiencies of the UCSPD.

Telecom photon input process	Efficiency
WDM + FC/APC connection	0.89
Fiber to waveguide coupling + propagation	0.436
**SFG process + converted photon output process**	**Efficiency**
SFG conversion efficiency	0.6
Waveguide to fiber coupling + FC/APC connection	0.67
**Filtering process + detection process**	**Efficiency**
U-bench with 2 bandpass filters	0.87
Si SPCM QE	0.6
Estimated total detection efficiency	0.0814

### 3.2 Detection of spontaneous parametric down-conversion photons

In this section, the performance of UCSPD in a practical single-photon experiment environment will be measured by comparing it with an InGaAs SPD. We will report measurement results detecting heralded single photons generated through a spontaneous parametric down-conversion (SPDC) process by comparing it with the reliable InGaAs SPD (AUREA, SPD_A_NIR_M2) and discuss it. Once to measure the detection efficiency of our UCSPD by comparison with the InGaAs SPD, which is the reference SPD, the detection efficiency of the reference SPD should be identified. The detection efficiency can be experimentally measured using a correlation between single counts and coincidence counts of the SPDC photon pair. The single counts can be readily measured by subtracting dark counts from the total single counts,

$$S_{\text{s}\left(\text{i}\right)}=S_{\text{total}}-D_{\text{s}\left(\text{i}\right)}=\mu \alpha_{\text{s}\left(\text{i}\right)},$$

where *S*_s(i)_, *S*_total_, *D*_s(i)_, *μ*, and *α*_*s*(*i*)_ represent the signal (idler) single counts subtracted the dark counts, the total single counts, the signal (idler) dark counts, the mean photon-pair number, and the total signal (idler) detection efficiency including all optics transmissions and SPD detection efficiency, respectively. Similarly, net coincidence counts also can be measured by subtracting accidental coincidence counts from total coincidence counts,

$${\text{CC}}_{\text{net}}={\text{CC}}_{\text{total}}-{\text{CC}}_{\text{accidental}}=\mu \alpha_{s}\alpha_{i},$$

where CC_net_, CC_total_, and CC_accidental_ refer to the net coincidence counts subtracted the accidental coincidence counts, the total coincidence counts, and the accidental coincidence counts, respectively. Following these two measured counts, the InGaAs SPD’s detection efficiency, including all optics transmissions, can be identified by,

(2) $$\alpha_{\text{s}\left(\text{i}\right)}=\frac{{\text{CC}}_{\text{net}}}{S_{\text{i}\left(\text{s}\right)}}.$$

**Figure 8:**
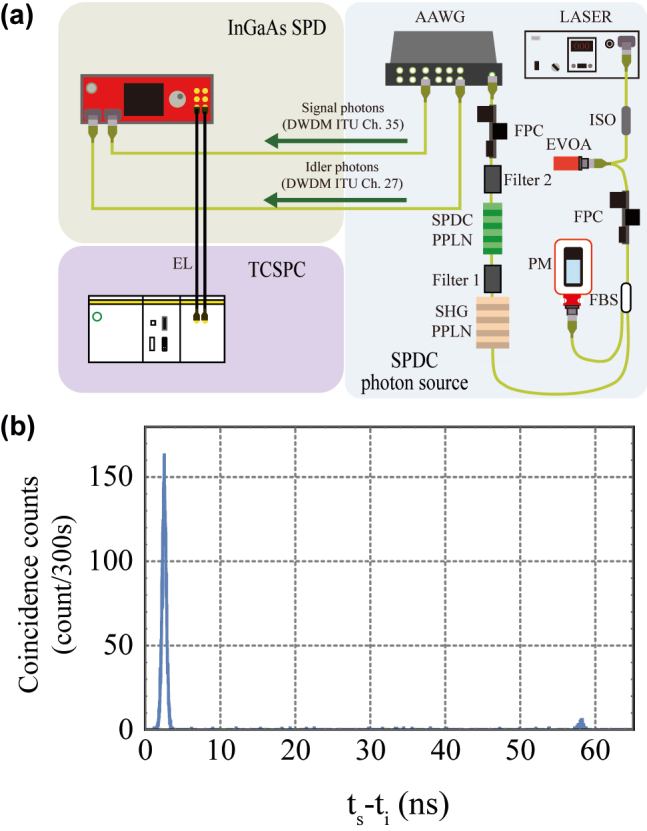
(a) Schematic optical setup diagram to define the InGaAs single-photon detector (SPD) detection efficiency with the time-correlated single photon counting (TCSPC). (b) Spontaneous parametric down-conversion photon-pair correlation measurement result, where *t*_s(i)_ represents signal (idler) photon detection time. LASER: pulsed LASER, EVOA: electrical variable optical attenuator, AAWG: athermal arrayed waveguide grating, SPDC PPLN: spontaneous parametric down-conversion PPLN (waveguide), SHG PPLN: second-harmonic generation PPLN (bulk), filter 1: 1550 nm block filter, filter 2: 775 nm block filter, EL: electric line.

Figure 8(a) is the experimental outline for measuring the InGaAs SPD’s detection efficiency with the heralded single-photon source. We used the heralded single-photon source using the type-0 SPDC process in the waveguide PPLN pumped by the second harmonic of 18.04 MHz picosecond mode-locked fiber laser (Calmar Laser, FPL-02CTT). Our SPDC photon source has a broad intensity spectrum with a bandwidth of about 80 nm centered at 1552.52 nm, so when a wavelength division is performed with the athermal arrayed waveguide grating (100 GHz channel spacing, 40 Ch), 40 signals with the bandwidth of 80 GHz can be divided into the corresponding wavelengths. We used 27 and 35 DWDM ITU channels to make the SPDC photon pair. The signal and idler photons were detected by the two channels of the InGaAs SPD operating free-running mode (quantum efficiency 10% and dead time 50 μs). The time-correlated single photon counting (TCSPC, PicoQuant, picoharp 300) measures the photon-pair correlation between the signal and idler photons. Figure 8(b) shows the result of the coincidence counts measured for 300 s with the TCSPC when the single counts detected by the channel 1 and 2 detectors are 4549 and 4665 Hz with 1543 and 1288 Hz dark counts. The channel 1 and 2 detectors measured the photons of 35 and 27 DWDM channels. The first main peak refers to the total coincidence counts (CC_total_), and the side peak is the accidental coincidence counts (CC_accidental_). The average CC_total_ and CC_accidental_ are 21.09 and 0.57 Hz. Then, the channel 1 detection efficiency of 0.61% can be calculated by Eq. (2), which value is the total efficiency, including the whole process from the creation of the heralded photons to the detection. In addition, since the quantum efficiency of the InGaAs SPD is almost constant in the C-band region, it can be assumed that the detection efficiencies in the C band are the same.

**Figure 9:**
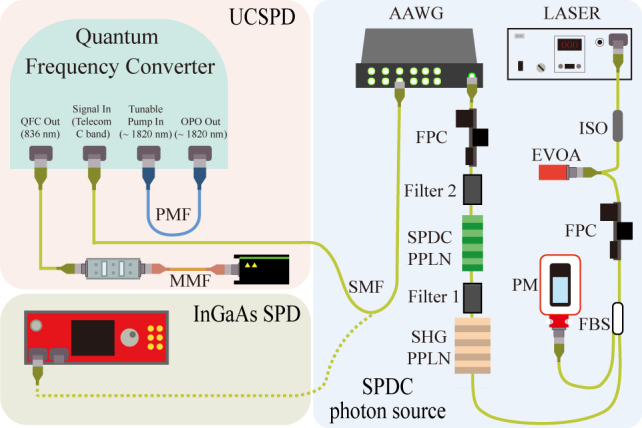
Schematic diagram of the optical setup for UCSPD performance test compared to the conventional InGaAs SPD using the heralded single-photon source.

The schematic of the experiment to measure UCSPD performance is shown in Figure 9. We used the signals of the nine wavelength-division channels in the DWDM ITU channel numbers 35, 37, 39, 41, 43, 45, 47, 49, and 51, and each of the signals is equally injected into both detectors. Then, we can measure the detection efficiencies of the UCSPD compared to the 0.61% detection efficiency of the InGaAs SPD obtained in the previous experiment.

**Figure 10:**
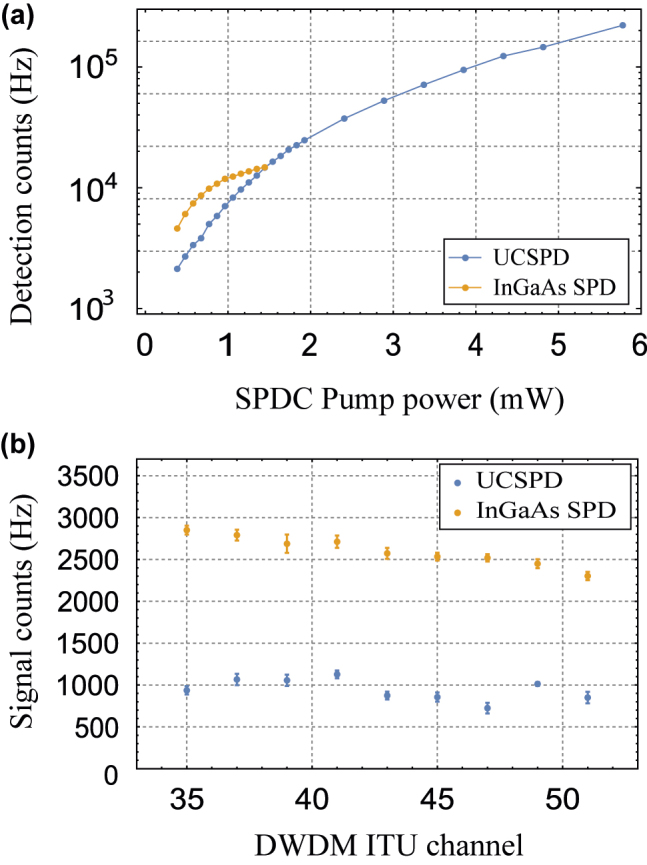
Up-conversion single-photon detector performance test results compared to the conventional InGaAs SPD. (a) Saturation limit measurement result. (b) Signal counts measurement result (signal counts = total counts − dark counts).

Figure 10(a) results from the detection counts of the 35 DWDM channel measured by both SPDs according to the pump laser power injected into the second-harmonic generation PPLN. When we show the result, it is confirmed that saturation is dominant from about 12,000 Hz when the InGaAs SPD is operated in free-running mode (quantum efficiency 10% and dead time 50 μs) with the 18.04 MHz picosecond laser. The results of the InGaAs SPD were measured until 14,700 Hz to ensure SPD safety. On the other hand, since the saturation limit of the UCSPD follows the characteristics of the Si SPCM, it is confirmed that it is linear even at 220,000 Hz or more. Figure 10(b) presents both SPDs’ single counts of the nine-channel signals. We set the pump laser power of 385.2 μW and alternately measured the same nine signals by both SPDs. The detection efficiencies of the UCSPD can be relatively calculated based on 0.61% of the InGaAs SPD’s detection efficiency used as the reference. The UCSPD’s detection efficiencies come out to be 0.18%–0.26%. However, since these detection efficiencies include all transmissions of the experimental system, it depends on the experimental system, so to know only the performance of the SPD, it should be expressed as quantum efficiencies. If it is assumed that input coupling efficiencies of the InGaAs SPD and the UCSPD are the same, the quantum efficiencies of our UCSPD can be estimated at 2.9–4.2% with 1 kHz dark counts, based on 10% quantum efficiency of the InGaAs SPD. Various quantum efficiencies came out because they use various SFG processes when measuring the telecom photons of the different wavelengths. Furthermore, since the heralded single photons distributed by the athermal arrayed waveguide grating have 80 GHz bandwidth, the quantum efficiencies also were reduced when measured with the UCSPD, which has the same detectable bandwidth as the athermal arrayed waveguide grating.

## 4 Conclusions

In conclusion, we have developed the tunable UCSPD, detecting all telecom C-band photons. For further practical feasibility of telecom-band UCSPD, the quantum efficiency may be improved to >20% by applying the high coupling engineering technique [20] with higher pump power, and the Raman noises for detecting photons above 1550 nm also may be reduced by changing the PPLNs’ parameters.

As our detector used the tunable OPO pump laser with the type-0 SFG process, it can detect all telecom C-band photons with the 80 GHz filtering effect. Thus, our detector can measure photons selectively with the bandwidth of 80 GHz even when a signal with a broad wavelength band is received. In state-of-the-art quantum communication systems, entanglement-distribution protocols have been proposed using wavelength-division multiplexing systems [25], [26], [27]. These protocols divide the wavelength multiplexed signal temporally to connect all users and detect it by SNSPD, which has a fast detection speed for classifying all signals. In the realistic implementation, however, not all nodes can have SNSPD, and it is difficult to distinguish all signals to increase the number of users temporally. Moreover, it is challenging to construct a fully connected network through temporal discrimination when distributing time-bin entanglement [2] due to the temporal modes used in the time-bin mode. Therefore, if our UCSPD is utilized for wavelength-division multiplexing systems, the quantum resources that require SNSPD for all nodes can be saved, and further, even if the number of users increases, all signals can be distinguished spectrally. Therefore, we anticipate our tunable integrated all-fiber UCSPD paves the way for reducing quantum resources used in wavelength-division multiplexing systems to establish a real-world quantum network.
